# The years of life lost on cardiovascular disease attributable to ambient temperature in China

**DOI:** 10.1038/s41598-017-13225-2

**Published:** 2017-10-19

**Authors:** Guijie Luan, Peng Yin, Tiantian Li, Lijun Wang, Maigeng Zhou

**Affiliations:** 1 Shandong Center for Disease Control and Prevention, Jinan, China; 2 National Center for Chronic and Noncommunicable Disease Control and Prevention, Chinese Center for Disease Control and Prevention, Beijing, China; 3 National Institute for Environmental Health, Chinese Center for Disease Control and Prevention, Beijing, China

## Abstract

Few studies have examined the association between ambient temperature and years of life lost (YLL). We aim to explore the burden of cardiovascular disease attributed to non-optimum temperature in China. YLL provides a complementary measure for examining the burden of disease due to ambient temperature. Non-optimal temperature leads to the increase of YLL. The mortality of fourteen cities in China during 2008–2013 was included in this study. We used the Distributed Lag Non-linear Model (DLNM) to estimate the association between daily mean temperature and YLL, controlling for long term trends, day of the week, seasonality and relative humidity. The daily YLL varied from 807 in Changchun to 2751 in Chengdu, with males higher than females. Extreme high and low temperatures were associated with higher YLL. The attributable fraction (AF) to cold effect is from 2.67 (95%CI: −1.63, 6.70) to 8.55 (95%CI: 5.05, 11.90), while the AF to heat effect is from 0.16 (95%CI: 0.06, 0.26) to 2.29 (95%CI: 1.29, 3.19). Cold effect was significantly higher than heat effect on cardiovascular disease in both men and women and for different age groups.

## Introduction

As the frequency of extreme temperature increases, numerous researchers have investigated the effects of high or low temperature on mortality^1–4^, especially in cardiovascular diseases^5,6^. The temperature-mortality relationship was usually depicted as U, J or V-shaped with delay effect^7,8^. The curve of the association between temperature and mortality shows that there exists the minimum mortality temperature, higher or lower than the optimum temperature could increase the excess deaths. Lag days of high temperature were one or two days, but the effects of low temperature lasted several days or even weeks^3,7^.

Although most of the previous studies have examined the association between extreme temperature and mortality and assessed whether there was evidence of excess mortality during extreme weather, few of them focused on the burden of disease associated with temperature^9,10^. Mortality only considered the number of deaths, and failed to take the death age into account. However, the short-term mortality displacement could heavily affect the relative risks of temperature on mortality. It would be more rational to assess the effect of ambient temperature exposure using the estimates of years of life lost (YLL), which takes into account the life expectancy^11^.

YLL, as an important part of Disability Adjusted Life Years (DALY), is a measure of disease burden that uses the life expectancy^12^. Compared with the traditional measure of mortality, YLL gives more weight to deaths among younger people. We should pay more attention to the effects of extreme temperature on young people who have a longer life expectancy than the elderly. At present, YLL is regarded as a more precise indicator to evaluate the burden of disease^12^ and some new studies^10,13^ have used YLL as outcome to evaluate the effect of ambient temperature around the world.

Cardiovascular disease is a major chronic non-communicable disease, accounting for about 40% of total deaths in China. About 3.7 million people died of cardiovascular disease each year, resulting in a serious increase on the burden of disease^14^. The change of ambient temperature was associated with human blood vessels’ contraction and may cause fluctuation of blood pressure. Literature has shown the rapid changes of blood pressure are likely to cause cardiovascular disease^15^. The evidence of the association between ambient temperature and the burden of cardiovascular disease is scarce in China. We collected data from Chinese national mortality surveillance system and investigated the association between extreme temperature and YLL in fourteen major Chinese cities during 2008–2013.

## Results

Table 1 shows summary statistics of daily YLL and weather for the fourteen Chinese provincial capital cities during the study period. The daily number of deaths was highest in Beijing (214) and lowest in Changchun (52). Descriptive statistics on population in fourteen Chinese cities by gender and age are showed (Supplementary Table S1). The Daily YLL was highest in Chengdu (2751) and lowest in Changchun (807). The mean temperatures varied significantly in different cities, ranging from 5.1 °C in Harbin to 21.6 °C in Guangzhou.

**Table 1:** Descriptive statistics on mortality, latitude and weather in fourteen Chinese cities.

City	Period (year)	Daily deaths	Daily YLL	Mean temperature(°C)							Relative humidity (%)	
				Min	5th	Median	95th	Max	Mean	SD	Mean	SD
Beijing	2008–2013	214	2531	−12.5	−4.6	14.9	28.5	34.5	13.1	11.4	52	20
Changchun	2008–2011	52	807	−27.6	−18.4	8.7	25.0	30.4	6.2	14.5	61	15
Changsha	2008–2013	92	1282	−3.0	3.1	19.1	32.3	35.8	18.3	9.4	73	14
Chengdu	2008–2013	200	2751	−0.5	4.1	17.4	26.8	29.3	16.3	7.5	76	9
Guangzhou	2012–2013	125	1571	5.1	10.0	23.0	29.2	30.4	21.6	6.2	81	10
Harbin	2008–2013	166	2592	−28.0	−20.2	8.0	25.4	30.6	5.1	15.6	66	15
Hefei	2012–2013	96	1242	−2.9	0.9	18.4	31.4	34.4	16.6	9.9	73	15
Jinan	2011–2013	109	1398	−9.4	−3.1	16.3	29.3	33.0	14.4	11.0	55	20
Kunming	2008–2013	94	1413	−0.9	7.9	17.0	22.2	24.6	16.1	4.8	68	14
Nanjing	2008–2013	101	1141	−4.5	0.3	17.8	30.2	34.6	16.3	9.7	70	14
Shanghai	2008–2012	163	1808	−3.4	1.8	18.3	30.5	35.7	17.2	9.2	69	13
Shenyang	2012–2013	144	1782	−21.1	−16.1	9.8	26.1	28.4	7.7	14.1	69	15
Shijiazhuang	2012–2013	139	1907	−8.1	−3.5	16.3	29.2	33.1	14.0	11.3	57	21
Tianjin	2008–2013	183	2250	−14.1	−5.1	14.4	28.2	32.4	12.8	11.5	57	18

**Figure 1:**
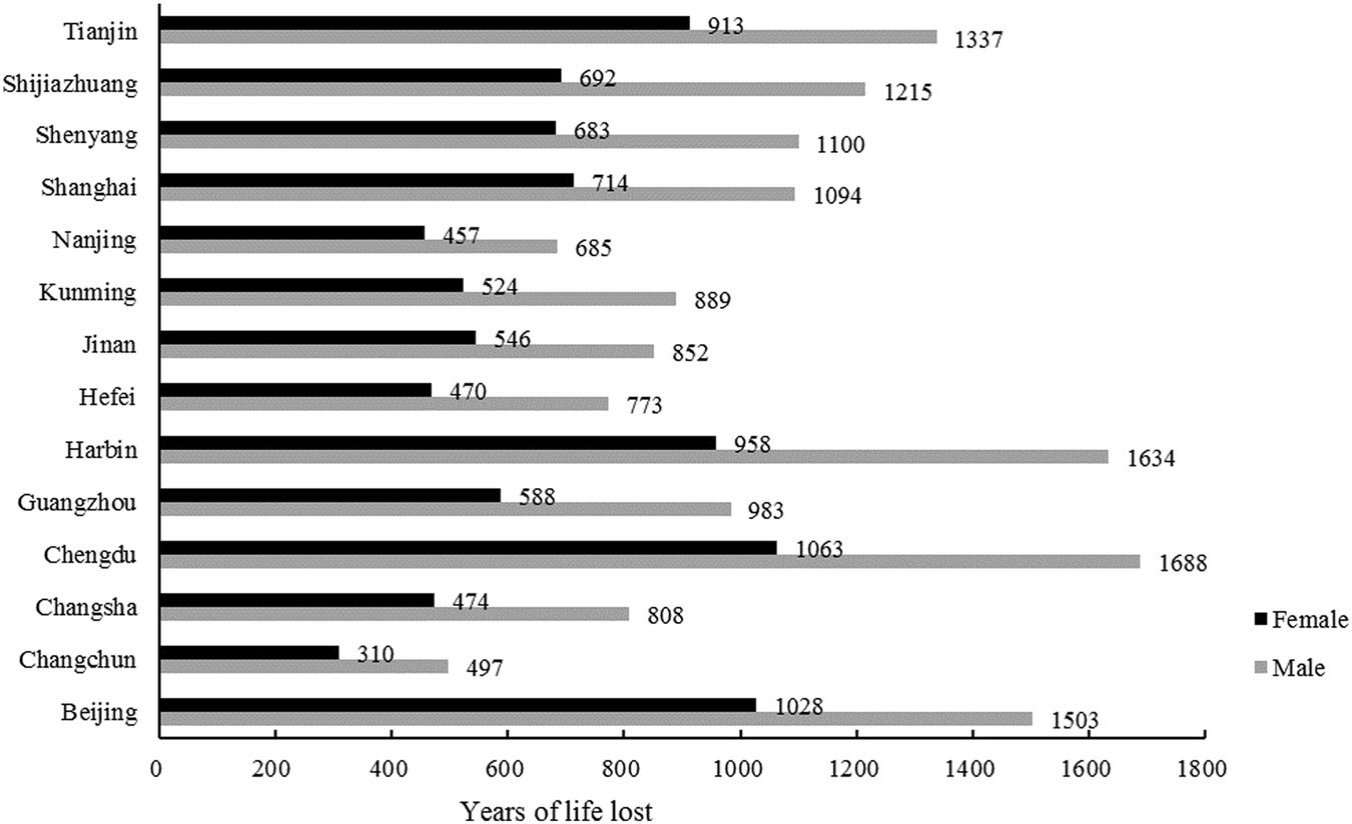
The years of life lost due to cardiovascular in fourteen Chinese cities by gender. Black bars represent Female, and gray bars represent Male.

Figure 1 shows the city-specific distribution of YLL by gender. YLL of cardiovascular disease was significantly higher in males than in females in all the fourteen cities, especially in Harbin and Chengdu.

**Figure 2:**
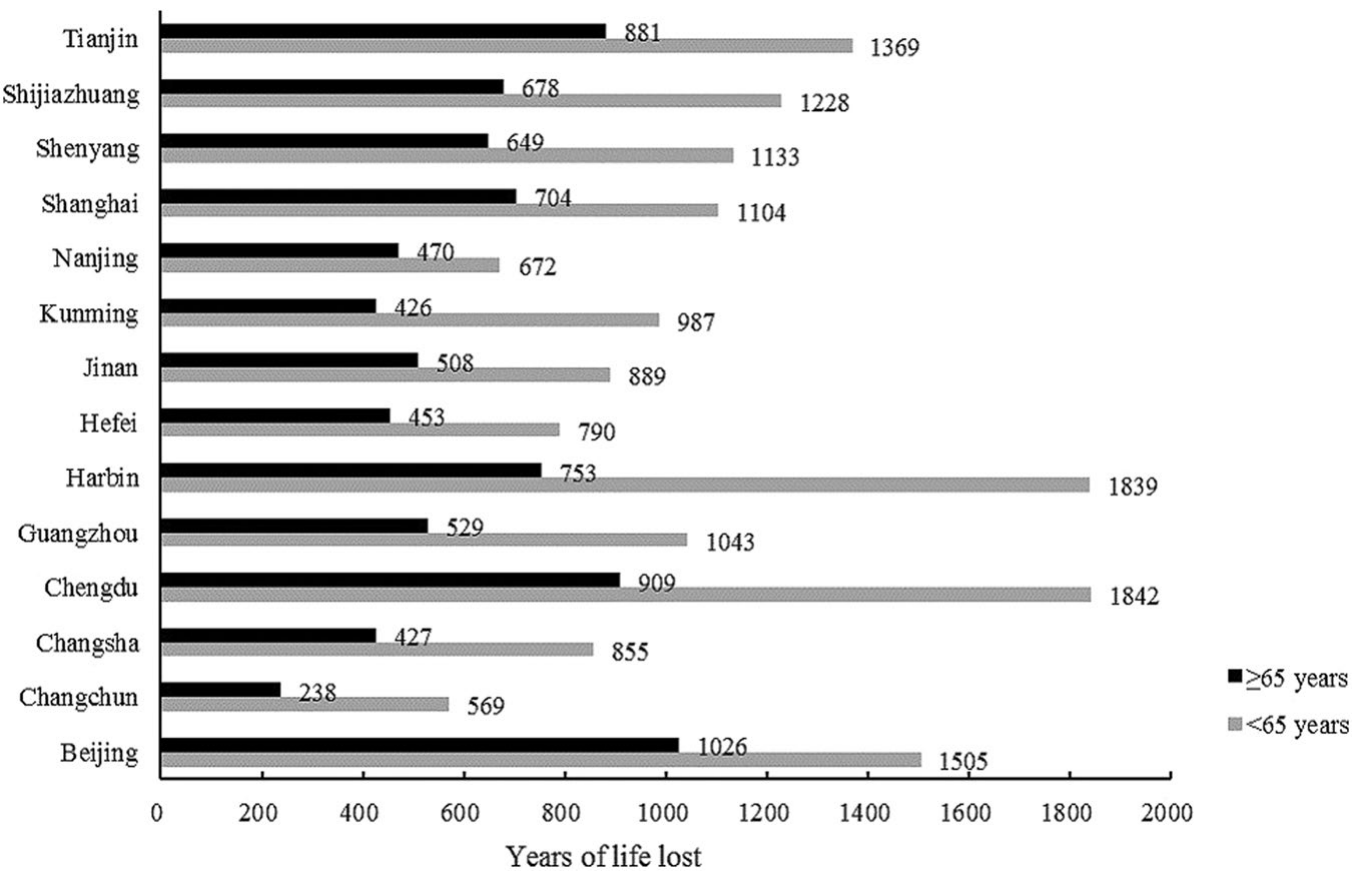
The years of life lost due to cardiovascular in fourteen Chinese cities by age. Black bars represent the age ≥65 years, and gray bars represent the age <65 years.

Figure 2 shows the city-specific distribution of YLL by age. YLL due to cardiovascular disease among those under the age of 65 years was higher than that in people aged greater than 65 years. The difference was two folds or more in cities like Harbin and Chengdu.

**Figure 3:**
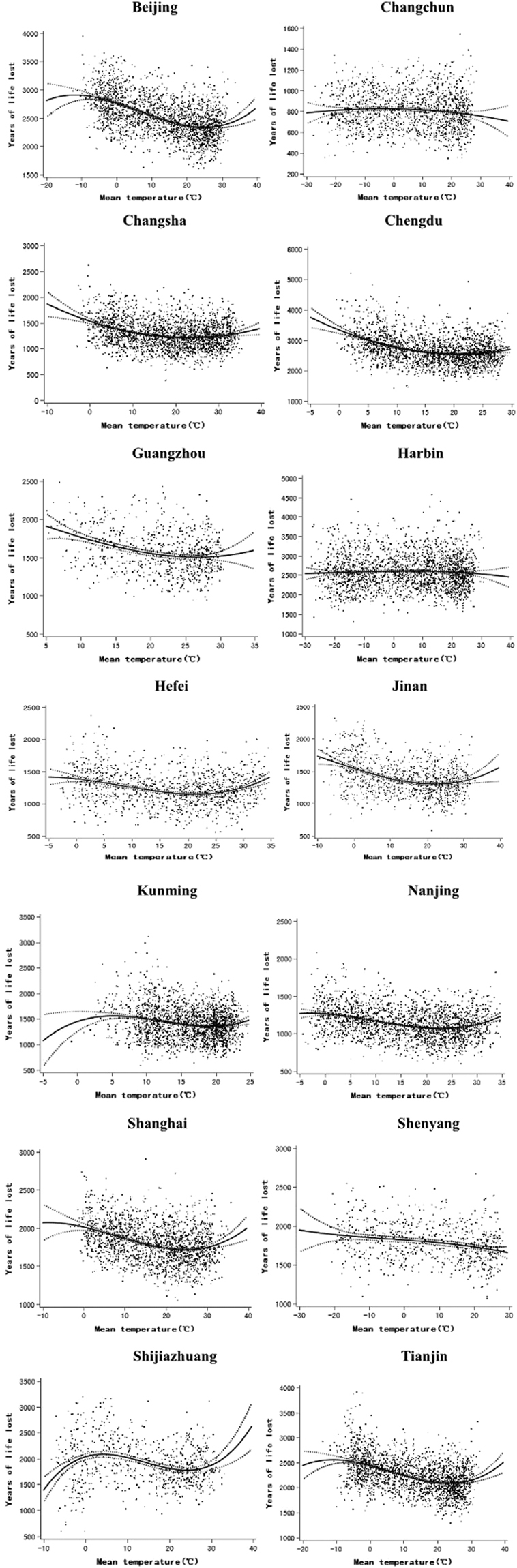
The Scatter plot of association between mean temperature and YLL in fourteen Chinese cities during the study period. The curves rooted in the cubic spline curve fitting.

As showed in Fig. 3, YLL decreased first and then increased with the increase in mean temperature. Extreme high and low temperatures were associated with higher YLL. These dots concentrated between 20 and 30 degrees, in other words, the optimum temperature was within this range. The effect of high temperature on mortality seems to be immediate but persists less than a week (Supplementary Table S2). However, the lag effect of low temperature is significant and can last two or three weeks. The overall cumulative effect of low temperature is significantly higher than high temperature (Supplementary Table S3).

Table 2 shows the estimated attributable fraction calculated as a separate component caused by low and high temperatures in each city. Overall, the total fraction of YLL varied significantly in different cities, with the highest attributable fraction in Changsha (10.42), and the lowest estimates in Hefei (3.52). The confidence intervals (CI) for Changchun, Hefei and Kunming were not significant. Cold effect was responsible for most of the burden of cardiovascular disease, while the fraction attributable to heat effect was small.

**Table 2:** The attributable fraction to cold and heat effect on YLL due to cardiovascular disease.

City	cold effect (95%CI)	heat effect (95%CI)
Beijing	6.80(3.19,10.07)*	1.37(0.75,1.93)*
Changchun	3.64(−2.35,9.31)	0.31(−0.57,1.09)
Changsha	8.13(4.68,11.10)*	2.29(1.29,3.19)*
Chengdu	7.27(3.94,10.39)*	0.31(−0.68,1.20)
Guangzhou	5.16(2.30,7.52) *	2.12(0.39,3.85)*
Harbin	6.65(0.02,12.44) *	0.87(−0.24,1.88)
Hefei	2.69(−1.25,6.23)	0.83(−0.12,1.61)
Jinan	2.67(−1.63,6.70)	0.96(0.02,1.88)*
Kunming	6.69(−1.96,13.59)	0.07(−0.12,0.23)
Nanjing	5.98(3.31,8.45)*	1.01(0.23,1.78)*
Shanghai	3.45(0.58,6.07)*	1.13(0.14,2.21)*
Shenyang	6.21(0.93,11.59)*	0.79(−0.68,2.08)
Shijiazhuang	5.62(0.02,10.50)*	0.16(0.06,0.26)*
Tianjin	8.55(5.05,11.90)*	1.56(0.88,2.17)*

*P < 0.05.

Table 3 shows the cumulative cold and heat effects in different cities by gender and age. Cold effect was significantly higher than heat effect for all subgroups. The total extreme temperature effect (heat effect and cold effect) was higher in males than in females in Beijing, Shanghai, Jinan, Hefei and Tianjin, whilst in the other 9 cities, the effect was higher in females than in males. The estimates of cold and hot-related YLL were higher in people age greater than 65 years, with the highest attributable fraction in Changsha, and the lowest estimates in Hefei.

**Table 3:** The attributable fraction to cold and heat effect on YLL due to cardiovascular by gender and age.

City	Male		Female		<65 years		≥65 years	
	cold effect (95%CI)	heat effect (95%CI)	cold effect (95%CI)	heat effect (95%CI)	cold effect (95%CI)	heat effect (95%CI)	cold effect (95%CI)	heat effect (95%CI)
Beijing	11.12(6.05,15.78)*	1.17(0.58,1.71)*	4.63(0.93,7.91)*	1.96(0.36,3.44)*	6.81(1.38,11.77)*	1.00(0.34,1.70)*	6.82(3.56,9.86)*	1.74(1.21,2.32)*
Changchun	3.55(−4.82,10.51)	0.23(−0.57,1.08)	5.37(−2.57,12.08)	0.96(−0.38,2.19)	4.79(−8.97,15.85)	0.62(0.19,0.99)*	7.80(2.19,12.94)*	1.15(0.02,2.16)*
Changsha	8.69(4.67,12.36)*	1.88(0.78,2.95)*	6.07(2.54,9.35)*	4.12(1.53,6.54)*	6.42(2.43,9.86)*	1.89(0.46,3.26)*	10.61(7.48,13.59)*	2.85(1.50,4.04)*
Chengdu	6.39(2.30,10.22)*	0.53(−0.33,1.39)	10.22(1.19,18.02)*	0.01(−0.08,0.06)	6.66(2.58,10.56)*	0.23(−0.83,1.21)	8.29(1.12,14.75)*	0.18(−0.09,0.44)
Guangzhou	4.21(0.82,6.96)*	1.47(−1.00,3.96)	6.63(3.63,9.32)*	3.25(0.09,6.29)*	4.22(1.41,6.58)*	2.62(0.05,5.36)*	9.01(5.65,11.92)*	1.17(−0.25,2.34)
Harbin	4.43(−4.87,12.09)	0.39(−0.67,1.39)	7.55(0.46,13.93)*	1.59(0.37,2.77)*	6.60(−1.86,13.89)	0.85(−0.27,1.91)	7.57(2.08,13.24)*	0.93(0.12,1.88)*
Hefei	5.71(0.38,11.40)*	0.87(0.19,1.85)*	2.43(−1.82,6.45)	0.91(−1.38,3.00)	4.01(0.33,7.49)*	0.99(−0.19,2.09)	6.81(3.43,10.05)*	0.96(0.01,1.78)*
Jinan	5.65(0.14,11.25)*	0.78(0.14,1.68)*	1.49(−2.17,4.63)	1.09(−1.31,3.49)	1.85(−1.16,4.36)	0.91(−0.90,2.68)	3.65(−0.35,7.30)	2.53(1.77,3.25)*
Kunming	4.06(−3.35,10.91)	0.17(−0.16,0.46)	7.03(1.12,15.93)*	0.03(−0.19,0.09)	4.43(−3.87,11.25)	0.13(−0.18,0.38)	13.14(1.06,23.85)*	0.01(−0.11,0.12)
Nanjing	5.26(2.75,7.59)*	2.06(0.11,3.63)*	8.53(1.46,14.93)*	0.85(0.62,1.05)*	4.69(1.55,7.62)*	1.04(0.12,2.16)*	6.78(4.09,9.45)*	1.17(0.28,1.94)*
Shanghai	4.89(1.77,7.69)*	1.12(0.23,2.55)*	1.81(−1.31,4.64)	1.41(−1.27,4.27)	2.95(0.02,5.63)*	1.66(0.23,3.49)*	7.93(4.88,10.73)*	0.84(0.08,1.58)*
Shenyang	6.21(−1.78,12.58)	0.65(−0.56,1.85)	7.96(1.72,13.25)*	1.28(0.35,2.62)*	4.67(−1.66,10.91)	0.36(−0.12,0.79)	8.63(3.65,13.15)*	0.90(−0.40,2.15)
Shijiazhuang	5.54(−0.70,11.08)	0.24(−0.17,0.58)	5.56(−2.27,12.41)	0.45(0.25,0.64)*	4.81(−1.16,9.59)	0.02(−0.08,0.12)	5.38(−0.75,10.95)	0.38(0.04,0.70)*
Tianjin	9.66(4.72,14.06)*	1.33(0.62,2.00)*	7.17(4.07,10.33)*	2.45(0.98,3.82)*	7.07(1.90,11.88)	1.18(0.47,1.96)*	9.07(6.34,11.81)*	1.95(1.17,2.68)*

*P < 0.05.

## Discussion

In this study, we found that the association between ambient temperature and YLL was U-shaped curve, with a significant increase of YLL associated with low temperatures. The effect of low temperature on cardiovascular disease was higher than high temperature, and there exists the minimum value between ambient temperature and YLL of cardiovascular disease.

Current studies^16,17^ mainly focused on the impact of temperature on mortality, always biased by the fact that the abnormal temperatures have a greater impact on older people^18^. We used YLL as the outcome variable in this study, which fully takes into account the death weight of different age. It can be used to not only evaluate the disease burden of the temperature, but also to respond to the impact of health risk factors on the population. In measuring the health outcomes of extreme weather events, YLL representing the burden of specific diseases can act as a more concise and synthetic indicator than mortality, and therefore can convey more useful and straightforward information to decision makers.

In this paper, the effect of ambient temperature on the YLL of cardiovascular disease was analyzed by DLNM^19^. The results showed that the risk of temperature on YLL was nonlinear, high or low temperature could increase YLL. Unlike some studies^13,16,20^, the curves presented in this study were U-shaped (Supplementary Figure S1), instead of V or J-shaped. The difference was likely due to the discrepancy in population base and geographical region. Previous studies^21,22^ have shown that the impact of air temperature on the daily mortality of the population lagged behind, the lag time of high temperature and low temperature was different. The specific performance of high temperature effect was short, and the low temperature effect lasted for a long time, the relative risk of low temperature is significantly higher than high temperature. It was found that the association between temperature and YLL followed the same pattern, with no lag effect for high temperature and 7 days lag for the effect of low temperature, suggesting the high temperature warning should be early and responses should be rapid, while the low temperature should be paid attention to its longer effect and taking a relatively lasting response measures to reduce the adverse effects of low temperature.

As a part of the disability adjusted life years, YLL can be used to compare the burden of disease. The study shows that the temperature above or below the optimum temperature will cause the increase in YLL. The burden of disease caused by low temperature was significantly higher than that of high temperature, which was attributed to our definition. We define that the temperature below the optimum temperature belongs to low temperature and above the optimum temperature belongs to high temperature. At present, most of the studies^23–26^ are more concerned with extreme weather (heat waves and cold spell), because this kind of research can offer more intuitive understandings of the acute temperature influence on mortality, and to provide the basis for the formulation of policy, such as the definition of heat wave or cold spell and the high temperature allowance. Since there was only about one-fifth of AF caused by the extreme low and high temperature^27^, we should not only pay attention to the impact of extreme weather, but also the influence of non-optimum temperature.

The significantly higher YLL of cardiovascular disease in men found in the current study might be due to the poorer diet and lifestyle habits, more mental stress and other aspects of exposure in men compared with women^28^. There is no regular change in gender, which was inconsistent with the results of a study of the number of deaths^29^. Women’s life expectancy is higher than that of men, especially in the elderly. Therefore, the impact of temperature on women may be overestimated in the previous studies. With the age stratified analysis, it was found that the temperature impact on the people aged <65 years was lower than the people aged ≥65 years, namely, the elderly were more vulnerable populations of extreme temperatures, which were consistent with previous studies^30,31^. The main reason might be that the elderly people’s temperature regulation ability and the ability to adapt to the temperature become weak, and the elderly tend to have one or more common diseases at the same time.

Low temperature increased blood pressure and heart rate and exposure to low temperature in patients with cardiovascular disease may cause coronary spasms, chest pains, and even myocardial infarction. By contrast, exposure to high temperature leads to an increase in deprivation of body fluids and hypotension, and then increases the burden of heart. Cardiovascular disease has become the highest mortality chronic diseases, the burden of disease it caused has been gradually being acknowledged and emphasized. Environmental factors, especially ambient temperature and air pollution^32^, together with the lifestyle, exercise, diet and other risk factors^33,34^, play a vital role in the incidence and mortality of cardiovascular disease.

To our knowledge, this is the first study in China investigating the association between ambient temperature and the burden of cardiovascular disease measured by YLL. We utilized mortality surveillance data with proved good quality in a relatively large time span (2008~2013). We examined the association in fourteen major Chinese capital cities across different regions of China with significant temperature variations. Furthermore, we utilized YLL as an indicator of the burden of disease and explored the association between temperature and YLL.

We also have limitations. Firstly, the model does not control the effects of air pollution as confounding factors due to the lack of accurate matching of air pollution data for each day, such as PM_2.5_, NO_2_ or O_3_. We will investigate how to integrate air pollution measurements in subsequent versions of our model. The interaction between air pollution and temperature^35^, might have an impact on the results. Further studies should be undertaken ruling out the noise of air pollution. Secondly, we used the national life expectancy table rather than the city-specific life tables to calculate YLL because of the data availability. Although it may cause inaccuracy of YLL estimates, we believe it’s not likely to significantly change the results because the included capital cities have similar economic and development levels.

## Conclusion

In summary, this study provides evidence that exposure to non-optimum ambient temperature increases the burden of cardiovascular disease in China. Low temperature has a more harmful influence than high temperature, and low and high temperatures-associated YLL were higher in the elderly. In order to reduce temperature-related YLL, the government should take measures to protect vulnerable people, especially patients with chronic diseases.

## Material and Methods

### Data sources

**Figure 4:**
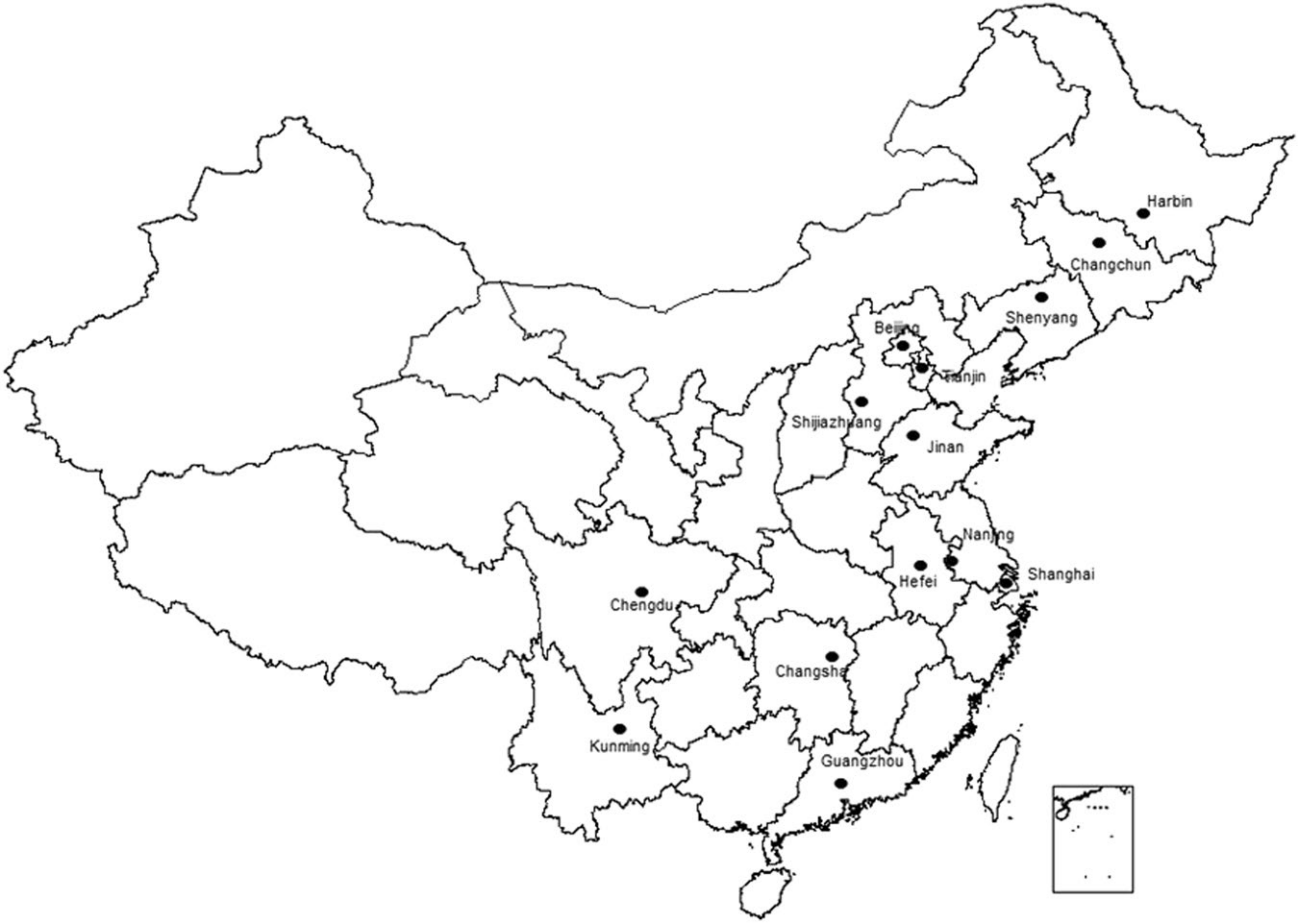
Location of 14 Chinese capital cities in this study. Black spots represent the locations of capital cities. This map was generated by ArcGIS software, version 10.1.

We collected daily cardiovascular mortality data of 14 Chinese cities (Fig. 4) during 2008–2013. The population of the cities ranged from 5.7 million in Hefei to 23 million in Shanghai (median 10 million in Shijiazhuang and 8.1 million in Shenyang). The mortality data of Guangzhou, Hefei, Shenyang and Shijiazhuang were from 2012 to 2013, Changchun’s mortality data were from 2008 to 2011, Shanghai’s data were from 2008 to 2012, Jinan’s data were from 2011 to 2013, and the other cities (Beijing, Changsha, Chengdu, Harbin, Kunming, Nanjing, Tianjin) were from 2008 to 2013. The code of underlying cause of death based on the 10th International Classification of Diseases (ICD-10) was used for deaths due to cardiovascular diseases(I00-I99). All data were classified by gender and age. The data were obtained from the China National Mortality Surveillance system^36^, which was administered by the Chinese Center for Disease Control and Prevention (China CDC). Detailed descriptions of the surveillance system and reporting procedures were published elsewhere^37^. In brief, all deaths occurred in the surveillance sites are required to be reported to the system. The underlying cause of death is coded by doctors and trained coders for death occurred in hospitals, while verbal autopsy is performed for deaths outside hospitals by village health workers or doctors at local hospitals^38^. Stringent quality control measurements are in place, including annual quality control meetings, staff training courses, development of regulations for death registration and regular site quality inspection.

The meteorological data for the same period were collected from the China Meteorological Data Sharing Service System (http://data.cma.cn/), which included daily mean temperature, minimum temperature, maximum temperature and relative humidity.

### Measurements of YLL

We calculated the YLL for each death (YLLi) and each day (YLLt) by the following formulas^13^:

1 $${\boldsymbol{YLLi}}{\boldsymbol{=}}{\boldsymbol{LEr}}\,{\boldsymbol{-}}(\,{\boldsymbol{A}}{\boldsymbol{-}}({\boldsymbol{ALB}}{\boldsymbol{+}}0{\bf{.5}}))$$

2 $${\boldsymbol{YLLt}}{\boldsymbol{=}}{\sum }_{{\boldsymbol{i}}{\boldsymbol{=}}1}^{{\boldsymbol{n}}}{\boldsymbol{YLLi}}\,$$

where *A* is the actual age at death for each individual, *LEr* is the remaining life expectancy at age *A*, *ALB* is the lower bound age from the life table. The national life table 2013 was obtained from the World Health Organization (WHO) (Supplementary Table S4). YLL was calculated by matching gender and age to the life table for each death. We calculated daily YLL by summing the YLL for all deaths on the same day. The sums were stratified by gender and age group (<65 and 65 + years).

### Statistical analysis

We applied the Distributed Lag Non-linear Model (DLNM) to estimate the impact of heat and cold temperature on YLL with the following formula:

3 $${\boldsymbol{YLLt}}{\boldsymbol{=}}{\boldsymbol{\alpha }}{\boldsymbol{+}}{\boldsymbol{NS}}\,({\boldsymbol{Time}},\,{\boldsymbol{T}}\ast {\bf{7}}){\boldsymbol{+}}{\boldsymbol{NS}}\,({\boldsymbol{Humidity}},\,{\bf{3}}){\boldsymbol{+}}{\boldsymbol{\gamma }}\mathrm{Dow}{\boldsymbol{+}}{\boldsymbol{\eta }}\,{\boldsymbol{Holiday}}{\boldsymbol{+}}{\boldsymbol{\beta }}\,{\boldsymbol{Tempt}}$$

where *t* is the day of observation; *YLLt* is the summing YLL at day *t*; α refers to the intercept; *NS* represents natural cubic spline; *NS(Time)* was used to control long-term trend and seasonality and the degrees of freedom (df) chooses 7, T is the time span; *NS(Humidity)* was used to control the confounding effects of humidity and the df is 3; *Dow* is day of the week and and *Holiday* is public holidays, both of them represented as categorical variables. *Tempt* refers to a two-dimensional natural spline for daily mean temperature with a lagged 14 days, considering the effect of low temperature on death and harvesting effect^20,39^. *γ*, *η* and *β* are coefficients for *DOW*, *Holiday* and *Tempt*. We modelled the exposure-response relationship with three internal knots placed at the 10th, 75th, and 90th percentiles of city-specific temperature distributions.

We used minimum mortality temperature (MMT) as the optimum temperature^39,40^. MMT was rooted in the best linear unbiased prediction of the overall cumulative exposure-response association in each city, which stands for a minimum mortality percentile between the 1st and the 99th percentiles. Exposure-response model needs MMT as the reference for calculating the attributable risk by re-centring the quadratic B-spline. We used the overall cumulative relative risk (RR) corresponding to each day’s temperature to calculate the attributable fraction (AF)^27^ of attributable YLL in the next 14 days for each day and each city. Confidence intervals (CIs) were calculated to assume a multivariate normal distribution of the best linear unbiased predictions of the reduced coefficients.

The heat and cold temperatures were defined as temperatures higher than the MMT and lower than the MMT, and then the attributable fraction to cold and heat effects on YLL due to cardiovascular was calculated.

Sensitivity analyses were conducted by changing df (6 and 10 per year) for time to control for season, df (4 and 6) for humidity and and the maximum lag days (7 days and 21 days) for daily mean temperature (Supplementary Table S5). All models were analyzed in R version 3.3.1.

### Data Availability

The datasets generated and analyzed during the current study are available from the corresponding author on reasonable request.

## Electronic supplementary material

Supplementary material
