# Supplementary material for: The years of life lost on cardiovascular disease attributable to ambient temperature in China
Source: Sci Rep. 2017 Oct 19;7:13531. doi: 10.1038/s41598-017-13225-2 (PMC5648808; doi:10.1038/s41598-017-13225-2)
Supplement: Supplementary file 1 — Supplementary material [file 41598_2017_13225_MOESM1_ESM.pdf]

The years of life lost on cardiovascular disease attributable to ambient temperature in China

Guijie Luan[1], Peng Yin[2], Tiantian Li[3], Lijun Wang[2], Maigeng Zhou[2*]

1. Shandong Center for Disease Control and Prevention, Jinan, China
2. National Center for Chronic and Noncommunicable Disease Control and Prevention, Chinese Center for Disease Control and Prevention, Beijing, China
3. National Institute for Environmental Health, Chinese Center for Disease Control and Prevention, Beijing, China

*Correspondence to:

Prof. Maigeng Zhou

National Center for Chronic and Noncommunicable Disease Control and Prevention

Chinese Center for Disease Control and Prevention

27 Nanwei Road, Xicheng District, Beijing, 100050 China

E-mail: maigengzhou@126.com

Tel: 86-10-63041471

Fax: 86-10-63041471

Supplementary Table S1. Descriptive statistics on population in fourteen Chinese cities.

City	Population	Males	Females	< 65 years	≥65 years
Beijing	20,692,994	10,681,009	10,011,985	18,859,093	1,833,901
Changchun	7,673,616	3,906,805	3,766,811	7,043,014	630,602
Changsha	7,186,269	3,663,621	3,522,648	6,539,159	647,110
Chengdu	14,192,512	7,219,177	6,973,335	12,791,128	1,401,384
Guangzhou	12,839,049	6,773,019	6,066,030	11,968,400	870,649
Harbin	10,607,607	5,363,098	5,244,509	9,715,043	892,564
Hefei	7,506,266	3,864,611	3,641,655	6,814,579	691,687
Jinan	6,944,139	3,484,129	3,460,010	6,304,167	639,972
Kunming	6,503,320	3,344,072	3,159,248	5,947,646	555,674
Nanjing	8,069,549	4,174,232	3,895,317	7,317,404	752,145
Shanghai	23,804,303	12,265,727	11,538,576	21,404,126	2,400,177
Shenyang	8,169,507	4,128,497	4,041,010	7,308,686	860,821
Shijiazhuang	10,383,677	5,306,366	5,077,311	9,537,377	846,300
Tianjin	14,131,507	7,570,738	6,560,769	12,904,015	1,227,492

Supplementary Table S2. The relative risks and their 95% confidential intervals of high temperatures on cardiovascular disease over lag0-14 days in fourteen Chinese cities.

City	Lag0	Lag0-2	Lag0-7	Lag0-14
Beijing	1.08(1.05,1.11)	1.17(1.13,1.21)	1.19(1.13,1.25)	1.20(1.12,1.29)
Changchun	1.12(1.04,1.20)	1.18(1.09,1.28)	1.21(1.07,1.37)	1.16(0.96,1.40)
Changsha	1.16(1.10,1.22)	1.23(1.16,1.29)	1.27(1.18,1.37)	1.28(1.15,1.43)
Chengdu	1.03(0.96,1.10)	1.03(0.94,1.13)	1.10(0.95,1.28)	1.13(0.89,1.45)
Guangzhou	1.03(0.98,1.09)	1.08(1.00,1.18)	1.07(0.94,1.23)	0.94(0.77,1.13)
Harbin	1.06(1.01,1.11)	1.05(1.00,1.11)	1.10(1.02,0.19)	1.10(0.99,1.22)
Hefei	1.12(1.04,1.22)	1.20(1.10,1.31)	1.06(0.94,0.21)	1.04(0.85,1.28)
Jinan	1.09(1.03,1.15)	1.19(1.11,1.28)	1.36(1.21,1.53)	1.38(1.15,1.66)
Kunming	1.02(0.98,1.07)	0.99(0.93,1.04)	0.99(0.90,1.08)	0.92(0.81,1.05)
Shanghai	1.06(1.01,1.11)	1.05(1.01,1.10)	1.08(1.02,1.15)	1.07(0.99,1.16)
Shenyang	1.07(1.00,1.15)	1.06(0.98,1.14)	1.08(0.95,1.21)	1.03(0.88,1.21)
Shijiazhuang	1.04(0.96,1.13)	1.02(0.92,1.14)	0.95(0.81,1.12)	0.91(0.69,1.21)
Tianjin	1.07(1.03,1.10)	1.12(1.07,1.16)	1.16(1.09,1.24)	1.18(1.08,1.30)

Supplementary Table S3. The relative risks and their 95% confidential intervals of low temperatures on cardiovascular disease over lag0-14 days in fourteen Chinese cities.

City	Lag0	Lag0-2	Lag0-7	Lag0-14
Beijing	0.86(0.81,0.92)	0.95(0.89,1.02)	1.19(1.08,1.32)	1.41(1.23,1.62)
Changchun	0.98(0.87,1.11)	0.97(0.85,1.12)	1.13(0.90,1.41)	1.14(0.74,1.75)
Changsha	0.85(0.78,0.91)	0.95(0.89,1.02)	1.33(1.20,1.47)	1.70(1.48,1.96)
Chengdu	0.95(0.82,1.09)	1.02(0.88,1.19)	1.26(1.03,1.54)	1.62(1.24,2.10)
Guangzhou	0.90(0.82,0.99)	1.01(0.91,1.12)	1.63(1.37,1.93)	1.97(1.53,2.55)
Harbin	1.00(0.90,1.10)	0.91(0.82,1.02)	1.08(0.85,1.37)	1.23(0.89,1.70)
Hefei	0.83(0.73,0.94)	0.84(0.73,0.96)	1.05(0.85,1.30)	1.47(1.09,1.99)
Jinan	0.82(0.75,0.90)	0.87(0.78,0.97)	1.00(0.84,1.19)	1.10(0.86,1.42)
Kunming	0.95(0.89,1.03)	0.99(0.91,1.08)	1.17(1.04,1.31)	1.38(1.19,1.60)
Shanghai	0.83(0.78,0.88)	0.88(0.82,0.94)	1.10(1.00,1.22)	1.27(1.10,1.46)
Shenyang	0.95(0.84,1.06)	0.97,0.83,1.13)	1.35(1.06,1.72)	2.03(1.46,2.84)
Shijiazhuang	1.00(0.83,1.25)	1.02(0.83,1.25)	1.12(0.68,1.84)	1.35(1.02,1.79)
Tianjin	0.89(0.82,0.96)	0.89(0.82,0.96)	1.22(1.08,1.38)	1.47(1.24,1.75)

Supplementary Table S4. National Life expectancy table 2013 for Chinese people from WHO.

Age	Female	Male
<1 year	77.0	74.1
0-4 years	76.8	74.0
5-9 years	72.9	70.2
10-14 years	68.0	65.3
15-19 years	63.1	60.4
20-24 years	58.2	55.5
25-29 years	53.4	50.7
30-34 years	48.5	45.8
35-39 years	43.7	41.0
40-44 years	38.8	36.2
45-49 years	34.1	31.5
50-54 years	29.4	26.9
55-59 years	24.9	22.5
60-64 years	20.6	18.4
65-69 years	16.5	14.6
70-74 years	12.9	11.3
75-79 years	9.7	8.5
80-84 years	7.0	6.1
85-89 years	4.9	4.3
90-94 years	3.2	2.9
95-99 years	2.3	2.1
100+ years	1.7	1.6

Supplementary Table S5. Sensitivity analyses of estimating association between ambient temperatures and the YLL of cardiovascular disease. Sensitivity analyses investigated potential confounding by changing maximum lag for mean temperature and degrees of freedom (df) for covariates. To calculate the attribute fraction to cold and heat effect on YLL due to cardiovascular disease

	Heat effect (95%CI)	cold effect (95%CI)
Main model	1.37(0.75,1.93)	6.80(3.19,10.07)
Df for year:6	1.40(0.86,1.59)	6.57(3.08,9.67)
Df for year:10	1.45(0.91,1.93)	6.43(3.06,10.01)
Lag period: 7days	1.28(0.75,1.56)	6.16(2.38,8.59)
Lag period: 21days	1.52(1.08,2.11)	7.16(3.89,11.42)
Df for relative humidity:4	1.36(0.74,1.78)	6.82(3.18,10.08)
Df for relative humidity:6	1.36(0.75,1.82)	6.81(3.17,10.07)

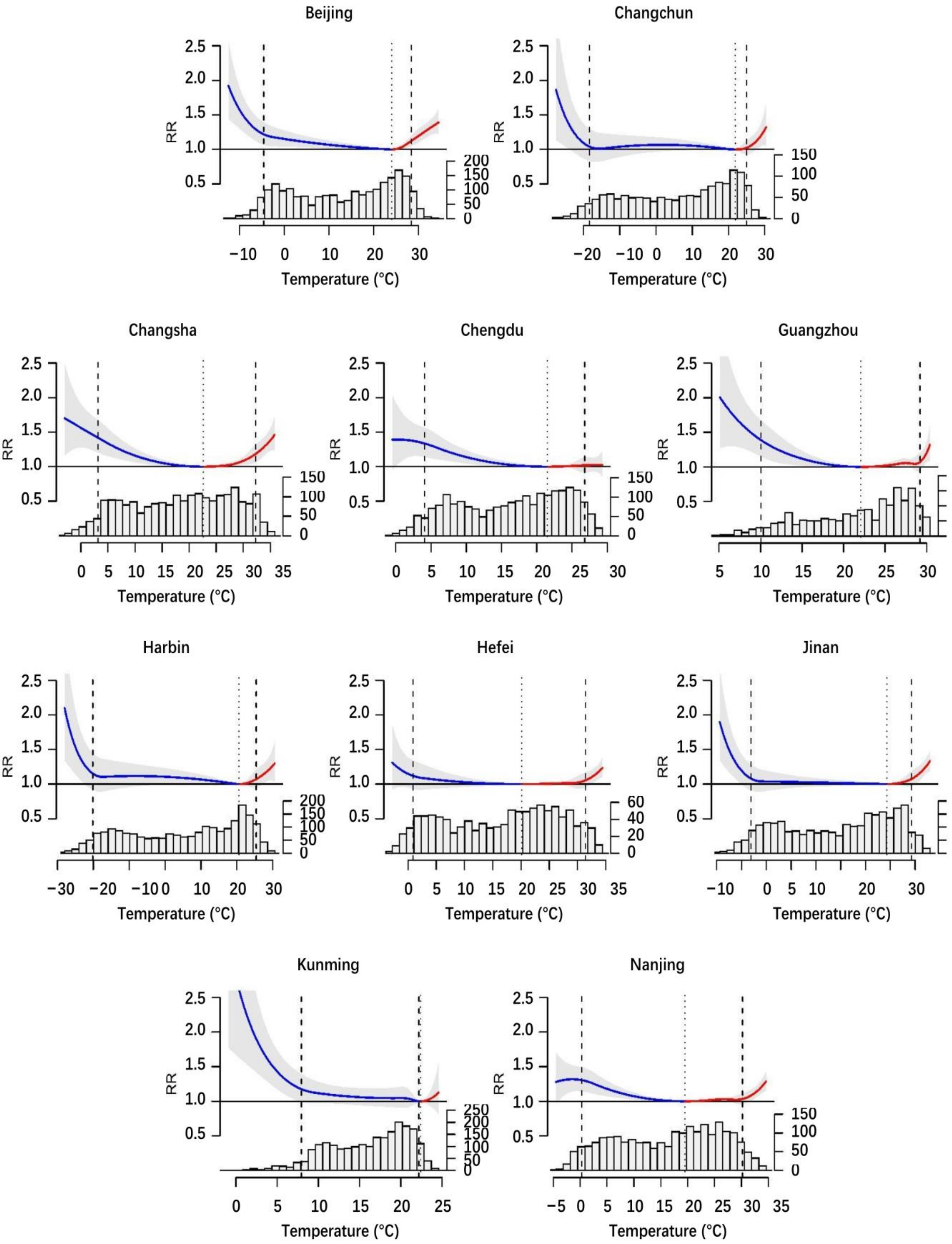
Beijing
Changchun
Changsha
Chengdu
Guangzhou
Harbin
Hefei
Jinan
Kunming
Nanjing
RR
Temperature (°C)

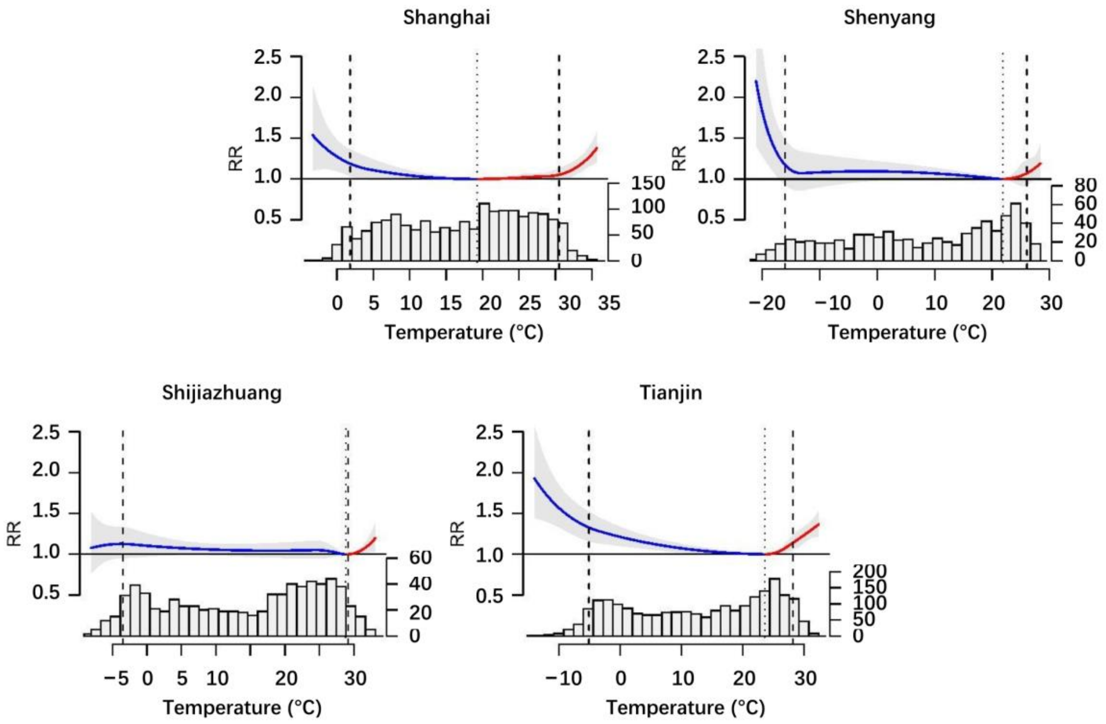

Supplementary Figure S1. The Relative risk of temperature and cardiovascular disease across lag0–14 in fourteen Chinese provincial cities during 2008–2013. The association between mean temperature and YLL is U or J shaped. The left Y-axis represents the relative risk (RR), the right Y-axis represents the daily YLL, the X-axis represents mean temperature. The curve represents the association between mean temperature and YLL, the histogram represents the distribution of YLL according to mean temperature.
